# Epibenthic and mobile species colonisation of a geotextile artificial surf reef on the south coast of England

**DOI:** 10.1371/journal.pone.0184100

**Published:** 2017-09-19

**Authors:** Roger J. H. Herbert, Ken Collins, Jenny Mallinson, Alice E. Hall, Josephine Pegg, Kathryn Ross, Leo Clarke, Tom Clements

**Affiliations:** 1 Bournemouth University, Department of Life and Environmental Sciences, Faculty of Science and Technology, Talbot Campus, Poole, Dorset, United Kingdom; 2 School of Ocean and Earth Science, University of Southampton, National Oceanography Centre, Waterfront Campus, European Way, Southampton, United Kingdom; 3 University Centre Sparsholt, Sparsholt, Winchester, Hampshire, United Kingdom; 4 British Trust for Ornithology, Thetford, Norfolk, United Kingdom; University of Sydney, AUSTRALIA

## Abstract

With increasing coastal infrastructure and use of novel materials there is a need to investigate the colonisation of assemblages associated with new structures, how these differ to natural and other artificial habitats and their potential impact on regional biodiversity. The colonisation of Europe’s first artificial surf reef (ASR) was investigated at Boscombe on the south coast of England (2009–2014) and compared with assemblages on existing natural and artificial habitats. The ASR consists of geotextile bags filled with sand located 220m offshore on a sandy sea bed at a depth of 0-5m. Successional changes in epibiota were recorded annually on differently orientated surfaces and depths using SCUBA diving and photography. Mobile faunal assemblages were sampled using Baited Remote Underwater Video (BRUV). Distinct stages in colonisation were observed, commencing with bryozoans and green algae which were replaced by red algae, hydroids and ascidians, however there were significant differences in assemblage structure with depth and orientation. The reef is being utilised by migratory, spawning and juvenile life-history stages of fish and invertebrates. The number of non-native species was larger than on natural reefs and other artificial habitats and some occupied a significant proportion of the structure. The accumulation of 180 benthic and mobile taxa, recorded to date, appears to have arisen from a locally rich and mixed pool of native and non-native species. Provided no negative invasive impacts are detected on nearby protected reefs the creation of novel yet diverse habitats may be considered a beneficial outcome.

## Introduction

With increasing development of coastal infrastructure there is a need to determine the ecological impact of artificial structures, how their colonisation compares with natural reef habitats and whether developing assemblages are likely to interact with existing biodiversity [1–3]. This is especially important in the vicinity of protected areas and where new structures consist of novel materials, are of unique design, unusual location and have multifunctional objectives that might generate attributes and disturbances not usually observed in the natural environment. Throughout the colonisation process, the arrival, establishment and replacement of species has the potential to interact with natural and other artificial habitats at local and regional scales. Structures may attract spawning adults, become nursery areas for juveniles [4,5] and the output of propagules could theoretically have an impact on dispersal between natural and artificial habitats [6]. A higher regional diversity of species and assemblages resulting from a wide variation of natural and artificial habitat may be considered beneficial by conferring a degree of resilience to a wide range of disturbances [7]. Yet of concern is the establishment of invasive species and the extent to which structures might provide stepping stones for their colonisation and spread [8–11], especially to nearby protected sites. This risk might be greater in the proximity of harbours and ports, which are known hot spots for invasive species [9].

Our aim was to investigate the colonisation and contribution to regional biodiversity of Boscombe Artificial Surf Reef (ASR), a novel structure deployed in shallow water (0–5 m Chart Datum) on the south coast of England. In the northern hemisphere, most studies of artificial reef colonisation have occurred in the Mediterranean, as relatively few Artificial Reefs (AR) have been constructed in the NE Atlantic and North Sea [12,13]. The concept of ‘succession’ as a progressive, directional and often predictable change in the colonisation of biological communities through species replacement over time is well established in ecology [14,15]. However patterns of colonisation and succession in different habitats and contexts vary and can be dependent on the type and morphological complexity of the substratum [12,16–19] and the temporal coincidence between availability of propagules and space for settlement [20]. The magnitude of recruitment success and establishment of early colonists may then have an impact on later-arriving species, depending on the extent to which they are facilitated, tolerated or inhibited through interspecific and intraspecific interactions [14,21,22]. The type and abundance of consumers that may remove early colonists is also influential [20,22,23], as is the extent of physical disturbances that can create patches of space by removing some or all epibiota [24]. This can result in mosaics of different assemblages composed of early and late colonists which vary spatially and temporally; the persistence of early and late colonists following disturbances may alter the direction of further community development [15].

As the ASR is in very shallow water and the upper section is exposed on low spring tides we were guided by examples of colonisation and succession from rocky shores in addition to subtidal natural and artificial reefs. A general view of the colonisation of hard bottom substrata in temperate regions [22,25,26] is that early colonists of bare space, which include diatoms and cyanobacteria and opportunistic species such as foliose and filamentous algae, are later replaced by perennial algae with upright and complex growth forms. In contrast to early opportunistic species, middle and late colonisers generally have a larger body size and are better competitors. However, early colonisation can be highly unpredictable and dependent on timing and the period of exposure of bare surfaces [27,28]. Understanding how species functional traits vary over the course of succession is weak. However on rocky shores the transition is generally from opportunistic ephemeral algae with little variation in traits to a more functionally diverse mature community [25].

Narrowneck geotextile artificial reef on the sub-tropical east coast of Australia, which, like the ASR was not primarily constructed to enhance local biodiversity, had over a three year period become colonised by macroalgae, mobile invertebrates, sea turtles and fish [29]. However due to the shallow, exposed and abrasive environment, combined with the novel geotextile substratum of the ASR, it was uncertain how colonisation of the structure would proceed and whether this might become functionally diverse and provide ecosystem services such as enhanced fisheries and benefits to dive tourism and snorkelling.

The complexity of the structure is highly influential in determining the diversity and type of assemblages [30–33]. At Narrowneck artificial reef this was considered important in limiting the range of taxa within the colonising assemblage as it is dominated by horizontal surfaces with few crevices and overhangs; however in a few areas where these features did occur, different species were present including soft-corals and bryozoans. On the ASR, variation in complexity is mostly between vertical and horizontal surfaces. Previous work in Australia [34–37] has shown differences in assemblages on reefs and structures with vertical and horizontal surfaces with some species affected more by orientation than location, independent of whether habitats are natural or artificial. Variation in species and assemblages have also been shown on differently orientated tiles or blocks [38–40] including substrate specific effects [34,41].

Another important feature of the ASR is its shallow depth and seaward gradient between 0m and 5m below Chart Datum. Variation in temperate subtidal assemblages due to water depth has been a neglected area of research [42], yet has revealed significant differences in composition and structure [42,43]. In shallow water the attenuation of surface light available to macroalgae [44,45] and its consumption by grazers [46,47] is likely to be particularly influential. So the interaction between depth and surface orientation could confer important variation in habitat to the ASR.

Concerning the colonisation of the ASR we made the following predictions:

1. As with new surfaces on natural reefs, rocky shores and other artificial substrata, there would be increasing coverage of epibiota and replacement of early opportunistic colonists by different functional groups over time.
2. Surface orientation would influence the type of benthic assemblages found on the ASR.
3. There would be significant variation in assemblages with depth.
4. Fish and other mobile consumers with different behaviour or life history traits would be similarly represented over the summer period and evidence of breeding or territorial behaviours would so far be minimal.

There is important debate about the impact and value of artificial reef habitats and structures, whether communities become comparable with natural reefs in terms of species diversity and if artificial reefs (AR) can be potential mitigation for habitat loss [33,48–51]. Variation in assemblage structure between natural and artificial reef habitats are frequently attributed to the age and early successional stages of the structure [19,37,52], differences in substrata [18,53] topographic and structural complexity [33,48,54], degree of isolation from natural reefs [55] seasonal movements of predators [56] and differential recruitment patterns [57].

In comparing the ASR to surrounding natural and artificial habitats we predicted significant differences in variation of assemblages of major functional groups.

## Materials and methods

### Study area and Boscombe Artificial Surf Reef

All surveys were carried out with permission of Bournemouth Borough Council.

**Fig 1:**
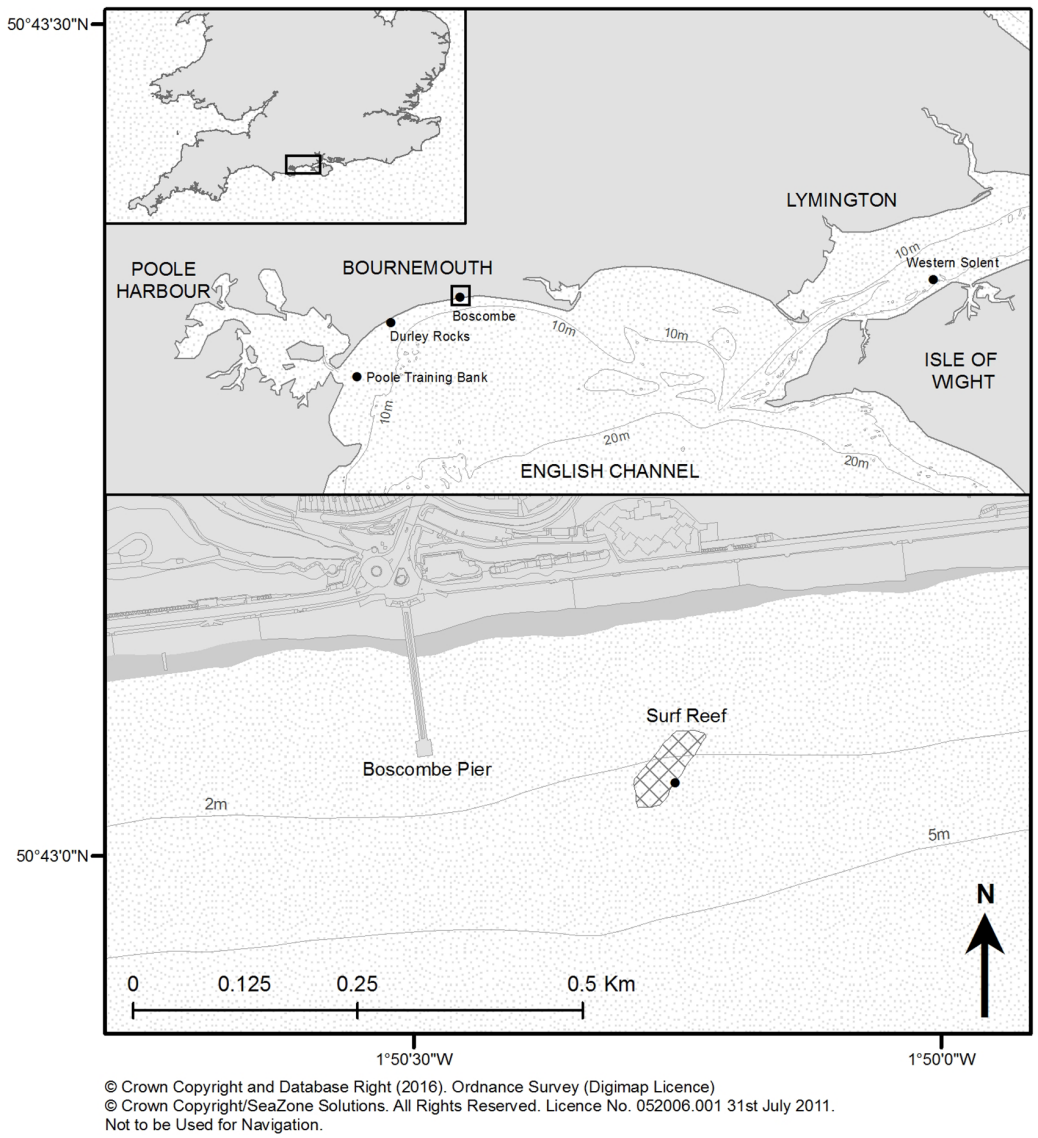
Study region. South coast of England showing location of the Boscombe Artificial Surf Reef (ASR) and other artificial and natural habitats.

The main study is centred at Boscombe within Poole Bay near Bournemouth on the central south coast of England (Fig 1) where there is considerable intertidal and subtidal infrastructure. However there are very few subtidal rocky reefs in the region, and those present are of small area in 6-16m water depth and mostly of sandstone or limestone rising 1-4m from the seabed. Owing to their scarcity and important assemblages, some of these reefs are protected areas. Nearby Poole Harbour is a busy port with ferry routes to the continent and has a considerable number of commercial and recreational craft. To the east of Poole Bay is the Isle of Wight, the Solent estuaries and the industrial ports of Southampton and Portsmouth. The coast is moderately exposed to south-west winds and annual sea surface temperature range is 7.5–18°C. The tidal range in Poole Harbour is 1.8m increasing to 3m in the western Solent. There is a bidirectional tidal flow, with the flood running east and the ebb to the west across the ASR and natural reefs. Close inshore, the sea bed in Poole Bay is medium sand, with more mixed sediments in the western Solent. There is a limited amount of trawling and netting for fish in Poole Bay and western Solent and some potting for cuttlefish (*Sepia officinalis*), brown crab (*Cancer pagurus*) and lobster (*Hommarus gammarus*) around the patch reefs. Recreational angling from charter vessels is very popular in the region.

Boscombe Artificial Surf Reef (ASR) is the first of its kind to be constructed in Europe. The original aim of the reef was to increase local tourism by creating better surfing [59–61]. It is located 220m offshore and 210m east of Boscombe Pier near Bournemouth in Poole Bay (Fig 1). Not only are purposely constructed artificial reefs relatively uncommon in northern Europe, the shallow location of the structure in the surf zone and use of novel geotextile material is unusual. The structure, which covers approximately 1 ha of the sea bed, is comprised of two sections consisting of 54 large sand-filled geotextile bags of 1-5m diameter and 15-70m length [59]. Construction of the bottom section commenced in August 2008 and the reef was completed in early November 2009. A propeller strike in March 2011 caused one of the bags in the top section to burst, which was lost and has not been replaced. Other repairs have been carried out following annual surveys. Unfortunately, the performance of the ASR as a surfing attraction has been very poor and its recreational use is minimal [61]. Details of other artificial and natural habitats sampled within the study are area shown in Table 1.

**Table 1:** Characteristics of study sites.

Location	Artificial/Natural	Depth (m CD)	Substrate Type	Notes
Artificial Surf Reef	Artificial	0–5	Geotextile sand bags	Completed 2009
Poole Training Bank	Artificial	0–2	Limestone blocks	Wall designed to direct the ebb and flow of currents to maintain navigable approach to Poole Harbour. Originally built in 1860, extended to 1300m in 1876 and 1500m in 1927 [58]
Boscombe Pier	Artificial	3–5	Concrete piles	Built 1861; reconstructed 1979–81.
Hamstead Ledge (Solent)	Natural Reef	12–16	Limestone	Potting for crab and lobster
Durley Rocks (Poole Bay)	Natural Reef	6–8	Sandstone	Potting for crab and lobster

### Monitoring the ASR colonisation

To test predictions (a), (b) and (c), monitoring the colonisation of the ASR utilised a combination of photography, underwater video using SCUBA diving and a mini-ROV (Remotely Operated Vehicle). Between December 2009 and October 2012 photos of epibenthic assemblages on horizontal and vertical surfaces were taken on the south-east side of the reef between 0-5m depth using an Olympus Mu digital camera with underwater housing (Table 2). In 2011, images were also obtained from ‘inclined surfaces’ at a depth of 2.5m. On each sampling occasion photos were taken haphazardly by the same person (KC) using a monopod to ensure a fixed distance from the substratum (field of view 16 x 24 cm) along haphazardly located transects from the sea bed to the reef surface. The time of each photo was retrieved and matched with depth and time data collected on dive computers [62]. Images from each photographed surface were placed in three depth categories 0–1.9m, 2–3.9m and 4–5.9m

**Table 2:** Number of photographs of the ASR. Photographs (16 x 24 cm) of ASR surface used for the analysis of temporal changes in epifauna and epifloral.

Year	Horizontal	Vertical	Total
16^th^ December 2009	NS	NS	**49**
15^th^ October 2010	33	44	**81**
16^th^ October 2011	28	28	**56**
7^th^ October 2012	26	35	**61**
**Total**	**87**	**107**	**247**

NS ~ orientation Not Specified.

Photography was also carried out haphazardly by volunteers in different areas of the ASR using a variety of camera systems and data was used qualitatively to compare with that recorded on the south-east side of the reef. A VideoRay Pro 4 ROV with smart-tether system and sonar was deployed from a boat at the ASR in November 2011 and August 2013 to qualitatively compare broad-scale coverage of epibiota from around the reef, to check for large scale disturbances to epibiota and damage, and the shoreward side that was least accessible by divers. All field work was carried out with permission of the local authorities.

To validate the identification of assemblages observed in photographs and compile a species inventory, samples of epibenthos were removed from the reef using a scraper and vacuum sampler, collected in a net bag and taken back to the laboratory for identification. Fauna samples were placed in 70% Industrial Methylated Spirit (IMS) and later identified to species level where possible. Other qualitative observations included the presence of sediments within crevices and the general condition of the sand bags.

### Image analysis

Each of the photographs were analysed using ‘Coral Point Count with Excel Extensions’ (CPCe) [63,64]. The percentage cover of benthic epifauna and flora from a broad range of phyla and functional groups was estimated from each photograph (brown algae, green algae, red algae, coralline algae, sponge, hydroids, anemones, polychaetes, gastropods, bivalves, barnacles, other crustacea, bryozoans, ascidians). These groups are known to be common or important occupiers of space within shallow rocky reefs in temperate regions and represent a variety of functional groups that include primary producers, filter and suspension feeders and grazers. It was found sufficient for 100 points to be positioned over each image to satisfactorily estimate percentage cover.

### Mobile consumers on the ASR

To test prediction (d) concerning the use of the ASR by mobile consumers, surveys of fish and invertebrates were undertaken between July and September 2013 using Baited Remote Underwater Video (BRUV). This is a cost-effective and non-destructive method of sampling suitable for the detection of a broad range of mobile species and functional groups in reef habitats [55,65–69]. The BRUV unit consisted of a single GoPro Hero 2 high-definition camera (www.gopro.com) with underwater housing fixed to a weighted aluminium frame. A plastic bait-arm, (20mm wide), attached to the base of the frame extended horizontally for 1m in front of the camera. Bait was retained in a plastic cage (5mm mesh) at the end of the bait arm and consisted of 100g of freshly chopped and crushed mackerel which was replaced for each deployment. Optimum soak times for estimations of species richness and abundance using BRUV vary [65,70] and few investigations have been carried out in northern Europe. Species accumulation curves produced from pilot studies at the ASR showed that footage of 20 mins duration was sufficient to obtain consistent estimates of abundance and species richness. Sampling was carried out using a single BRUV and necessarily restricted to daylight hours between 06:00–17:00 GMT. Six, 20 minute deployments were made during three periods in the summer; 2^nd^ July (Early Summer), 8^th^ and 21^st^ August (Mid-Summer) and 28^th^ August and 2^nd^ September (Late-Summer). On each sampling day, deployments varied spatially across the reef and at intervals of approximately 1 hour on both flood and ebbing tides to minimise any bias associated with the direction of the bait plume. Videos were examined on a PC using VLC media player. Fauna was identified to species level and the maximum number of individuals of each species seen in any frame during the 20 min deployment was recorded (MaxN) [65,67]. For quantitative analysis, only individuals seen within a field of view to a maximum distance of 1m (end of the bait-arm pole) were included due to variable visibility.

### Regional comparison with artificial and natural habitats

The epibiota of the ASR in 2012 was compared with two natural reef habitat sites: Durley Rocks (Poole Bay) and Hamstead Ledge (Western Solent), and two other artificial habitat sites: Boscombe Pier and Poole Training Bank (Fig 1). Between 2010 and 2012, using the same camera system described above, photographs were taken haphazardly on both horizontal and vertical surfaces along transects from the sea bed to the reef surface (Table 3). It was not possible to visit all sites in the same year due to weather and logistical constraints. At Boscombe Pier, photos were taken on vertical concrete pier legs only as these were the only accessible surfaces. At Poole Training Bank both horizontal and vertical surfaces were photographed when visited on foot during an extremely low spring tide.

**Table 3:** Number of photographs from other locations. Photographs analysed for benthic organisms on vertical and horizontal surfaces of the ASR and four reference locations.

Location	Date	Depth (m)	Aspect	Horizontal orientation	Vertical orientation	Total
ASR	7^th^ October 2012	0–5	South-east	26	35	61
Poole Training Bank	8^th^ October 2012	0–2	South -east	14	11	25
Boscombe Pier	16^th^ October 2011	3–5	South and east pilings	0	59	59
Hamstead Ledge	14^th^ August 2012	12–16	Mixed aspect	30	19	49
Durley Rocks	15^th^ September 2010	6–8	Mixed aspect	19	30	49
**Total**				**89**	**154**	**273**

### Data analysis

To test prediction (a), using combined data from all depths and surfaces, differences in the coverage of main functional groups from 2009 onwards were compared using non-parametric ANOVA. To test prediction (b), in 2010 and 2012 the coverage of the main functional groups on vertical and horizontal surfaces was compared separately using a Mann-Whitney U-test. In 2011, data from ‘inclined surfaces’ was also included and species groups were compared using non-parametric ANOVA. Variation in the overall assemblages present on the different surfaces was compared using ANOSIM [71].

To test prediction (c) concerned with differences due to depth variation, assemblages present in 2012 were compared using PERMANOVA [72]. ‘Depth’ and surface ‘Orientation’ were included as fixed factors, with an interaction term.

To test prediction (d), concerning the use of the ASR by mobile fauna, a group of species of known behaviour and life history traits [73,74] were selected and placed in four categories: (i) Adult pelagic species: European Sea Bass (*Dicentrarchus labrax*) and Grey mullet (*Liza* sp.); (ii) Adult territorial species: Corkwing wrasse (*Symphodus melops*) and Two-spotted goby (*Gobiusculus flavescens*); (iii) Migratory species: Spiny spider crab (*Maja squinado*), known to migrate in to local shallow waters and can form breeding aggregations or pods [75,76] and Black bream (*Spondyliosoma cantharus*) which migrates to shallower water in spring to spawn [77,78] and (iv) Juveniles: Black bream and Bib (*Trisopterus luscus*). Two-way ANOVA was performed on species counts (Max N) grouped by behavioural /life history traits across the summer period.

Canonical Analysis of Principal Components (CAP) ordination [79] was used to present variation in main epibenthic functional groups of nearby artificial and natural habitats and followed by ANOSIM.

## Results

### Colonisation of the ASR

**Fig 2:**
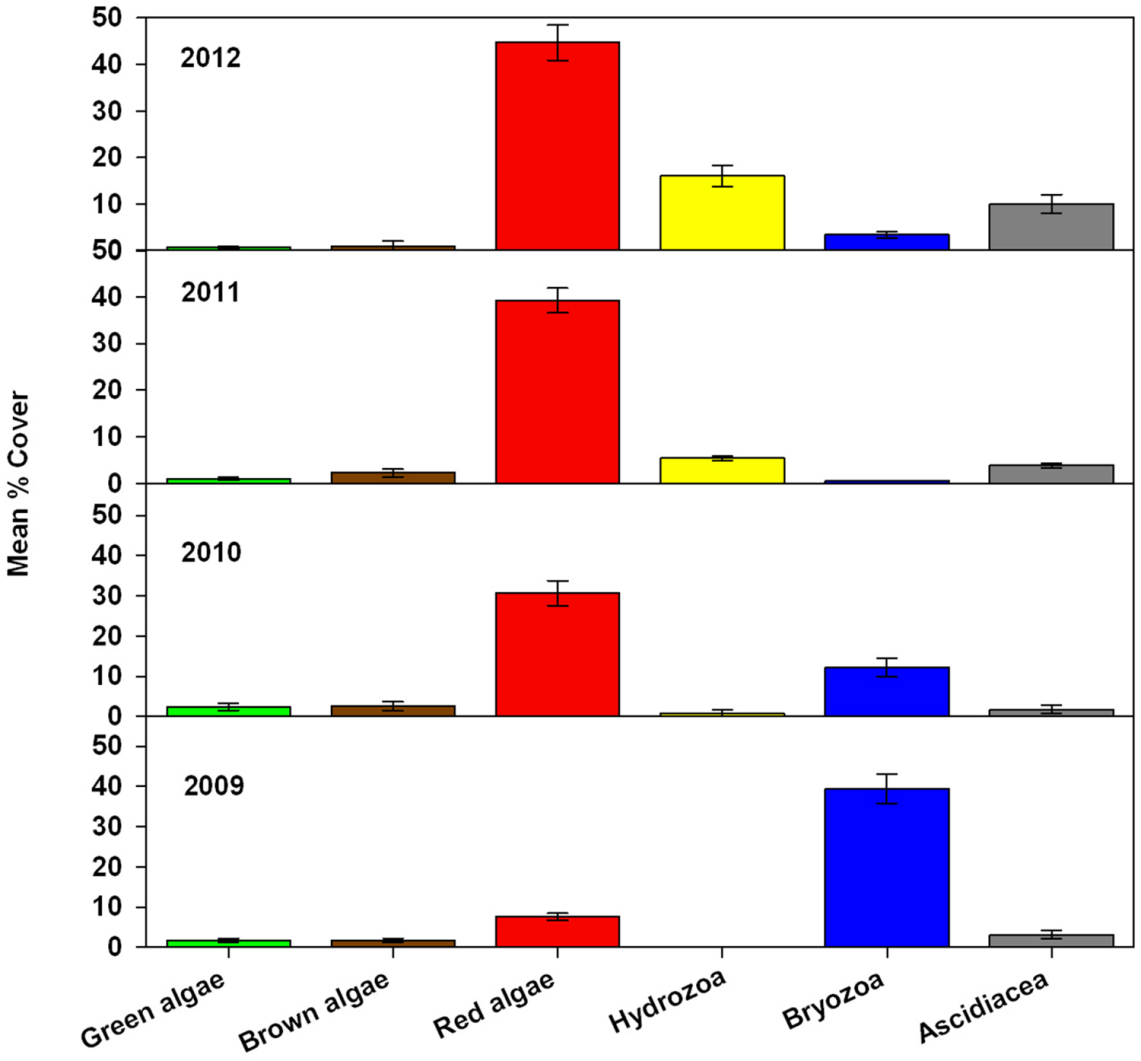
Mean cover of main groups of epibiota on the ASR 2009–2012. Data is mean of vertical and horizontal surfaces. Data from 2009 obtained in December, other years in October. Error bars show +SE.

From annual surveys between 2009 and 2014, including SCUBA and BRUV sampling, 180 taxa were recorded on the ASR, comprising 132 species of macro-invertebrates and fish and 48 species of algae. A list of species recorded on the ASR is presented in the Appendix. During 2008, prior to completion, diving contractors noted schooling fish over the ASR and green algae (*Ulva* spp.) was visible on the reef surface when exposed at extreme low water. The dominance of main algal and functional groups varied during the survey period (Fig 2). Between December 2009 and October 2010, the coverage of visibly ‘bare’ substratum reduced from 40–19% and over the same period, mean algal cover combining all surfaces increased from 12–56%. Differences in the coverage of the main algal groups were not always statistically significant (p<0.05) between years, although for brown and red algae these were evident when comparing data in 2009 and 2012. Inter-annual differences in the coverage of hydrozoa and bryozoan were mostly statistically significant, and ascidian coverage increased significantly between 2009 and 2012.

**Fig 3:**
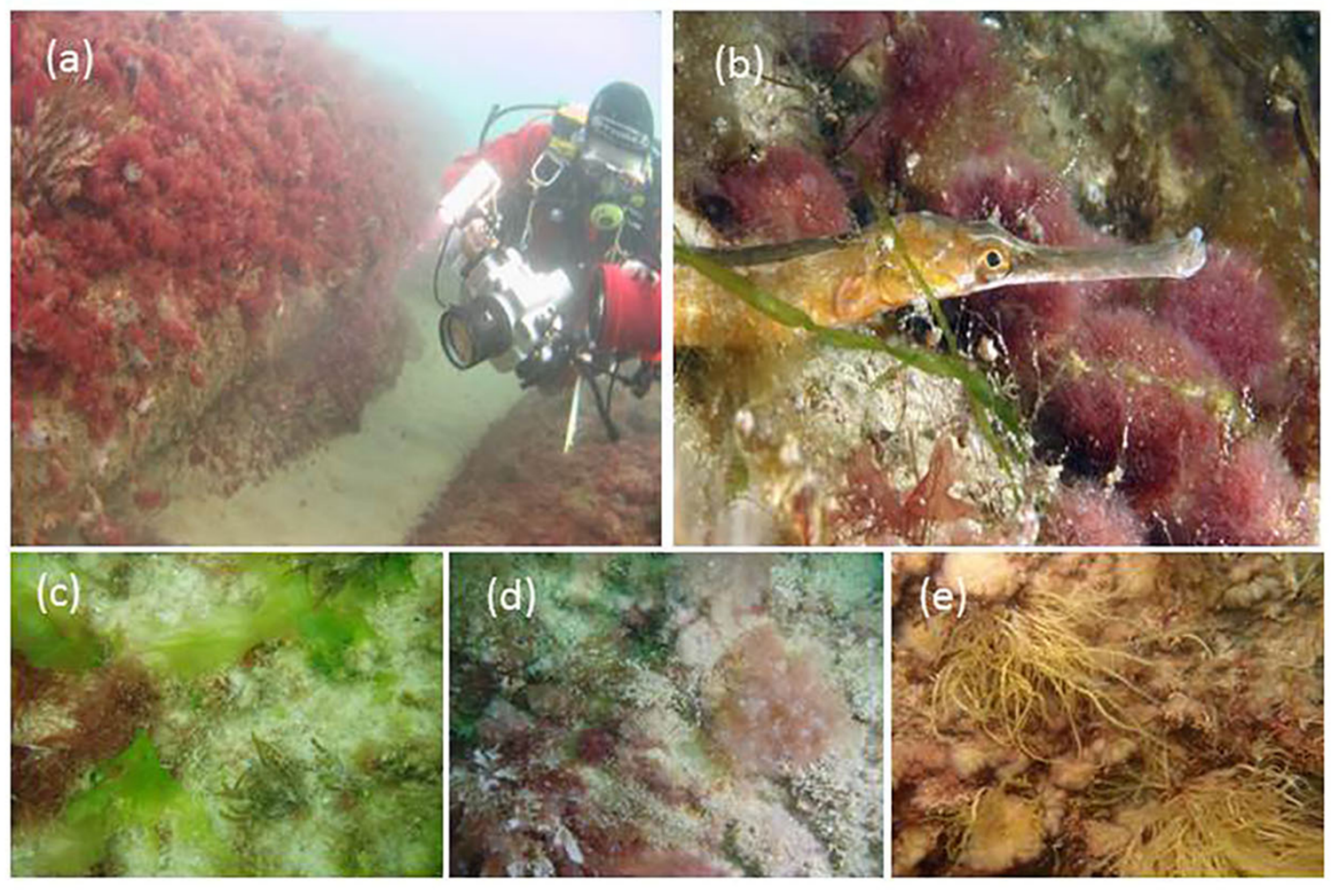
The Artificial Surf Reef. **(a)** South-eastern corner of the Artificial Surf Reef in June 2010. The upper red alga turf is dominated by non-native alga *Asparagopsis armata* (Falkenbergia stage). Close to the sea bed the reef is scoured, with scattered ascidians including *Styela clava*.; **(b)** Greater pipefish (*Syngnathus acus*) amongst red alga *Asparagopsis armata*. Photos (c-e) from point on south-east corner: **(c)** 4/6/2011 with *Ulva lactuca*, *Cladostephus spongiosus*, *Halurus flosculosus*; **(d)** 7/9/2012 with *Cryptopleura ramosa*, filamentous red algae and bryozoans) **(e)** 12/9/2013 with *Gracilaria gracilis* and *Asparagopsis armata* (Falkenbergia stage) (All photos KC, except photo (b) S Trewhela).

In December 2009, bryozoans, *Electra pilosa*, *Lissoclinum perforatum* and *Flustra foliacea* were dominant, yet during 2010 there was an increasing coverage of the brown alga *Cladostephus spongiosus*, green alga *Ulva lactuca* and red algae. By October 2010, 92 species of fauna and 32 algal species had been recorded on the ASR. Amongst these colonists were non-native species (NNS) including the alga *Sargassum muticum* and ascidians *Styela clava* and *Corella eumyota*. Eight fish species were recorded in 2010, including wrasse *Symphodus melops*, *S*.*bailloni* and pipefish *Syngnathus acus* (Fig 3) that are rarely recorded over the sandy habitats of Poole Bay. In 2011 a seasonal increase in red algae was recorded from 31–60% between June and October, dominated by the asexual (Falkenbergia) phase of the Indo-Pacific species *Asparagopsis armata* (Fig 3). Finer sediments including silt were evident in crevices between the sand bags from 2011–12 and the polychaete *Sabella pavonina* and bivalve *Venerupis corrugata* became more frequent in these habitats. Other molluscan fauna at this time was dominated by the gastropod *Rissoa parva*, whereas the blue mussel (*Mytilus edulis*), an important occupier of space on nearby structures, was uncommon. Apart from spiny spider crab *Maja squinado*, larger crustacea including lobster *Hommarus gammarus* and brown crab *Cancer pagurus* remained rare during the whole survey period. Eleven of the total 180 taxa recorded are thought to be non-native to Britain (~6%). However in 2012, some parts of the ASR, notably shallow and vertical surfaces had combined coverage of the alga *A*.*armata* and ascidian *S*.*clava* in excess of 50%.

### Effect of orientation and depth on the ASR assemblage

**Fig 4:**
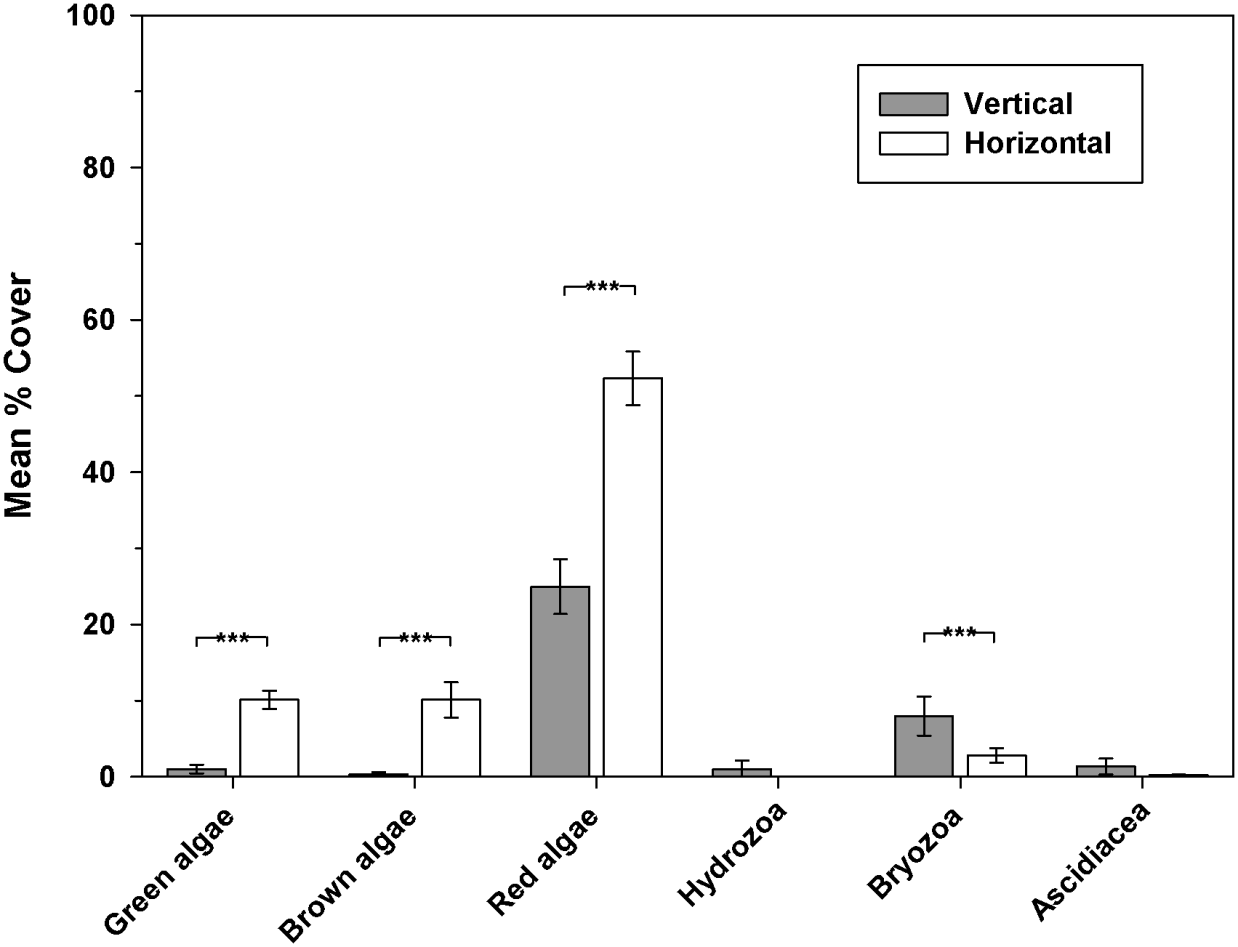
Epibenthic assemblages present on vertical and horizontal surfaces across all depths (0–5.9m) in 2010.

**Fig 5:**
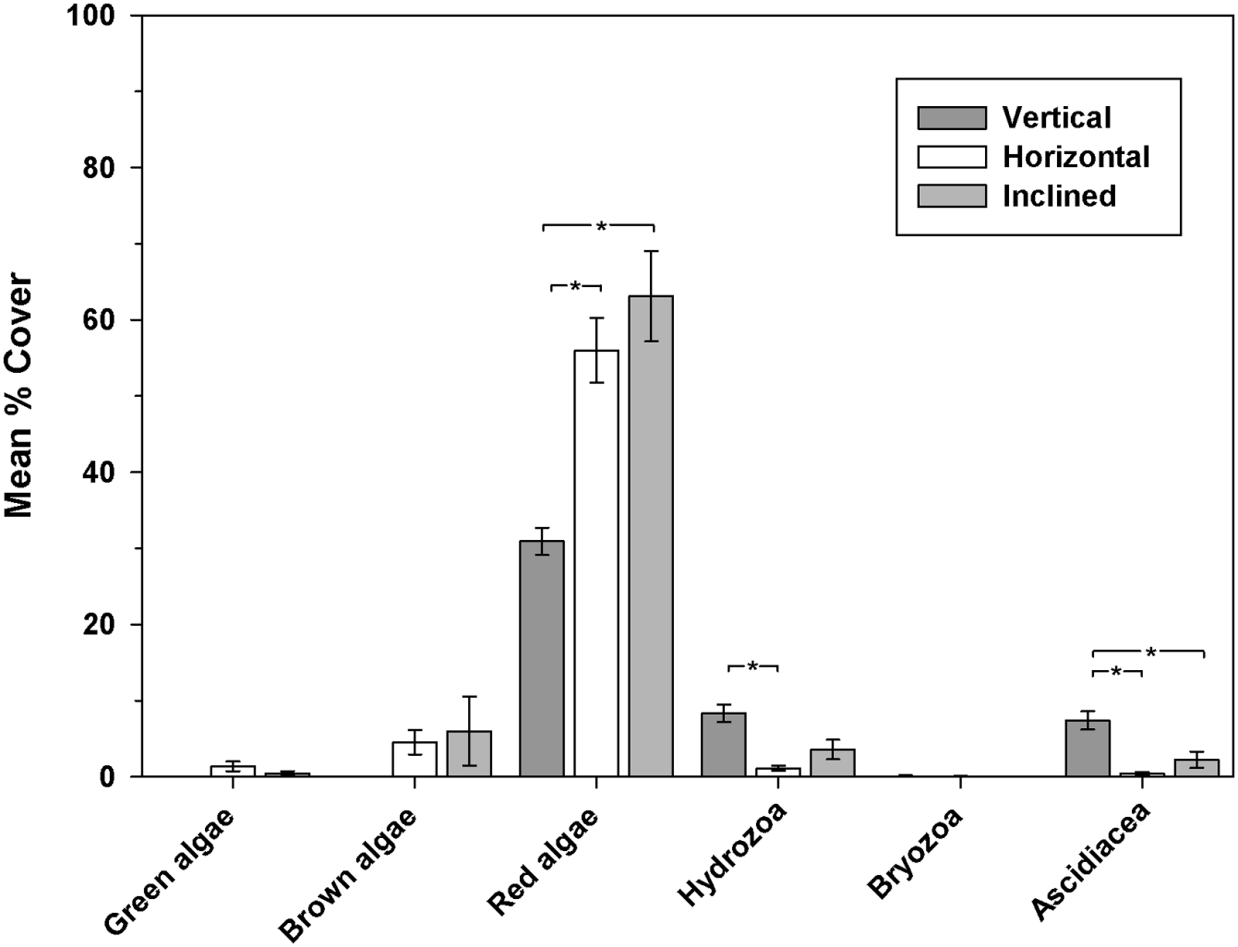
Epibenthic assemblages present on vertical, horizontal and inclined surfaces at depth 2–3.9 m in 2011.

Significant differences in coverage of all algal groups and bryozoans were apparent on different surfaces in 2010 (Fig 4). At middle depth, hydroids and ascidians became more prominent on the vertical surfaces in 2011 (Fig 5). Upper horizontal surfaces (0–1.9m) were dominated by red algae, green macroalga *Ulva* sp., and sand-scour tolerant brown algal species *Cladostephus spongiosus*. At middle and lower depths, most coverage was by the non-native red alga *A*. *armata*. The top of the reef lies within the shallow sub-tidal depth range in which non-native *S*.*muticum* became established locally.

Analysis of similarities (ANOSIM) showed a significant difference between horizontal and vertical assemblages within the 2–3.9m depth band in 2010 (R = 0.55, p = 0.0001). In 2011 a significant effect of orientation on assemblages within the 2–3.9m depth range was also apparent (R = 0.58, p = 0.0001) with pairwise comparison tests indicating significant differences (p<0.05) between vertical and both horizontal and inclined surfaces.

**Fig 6:**
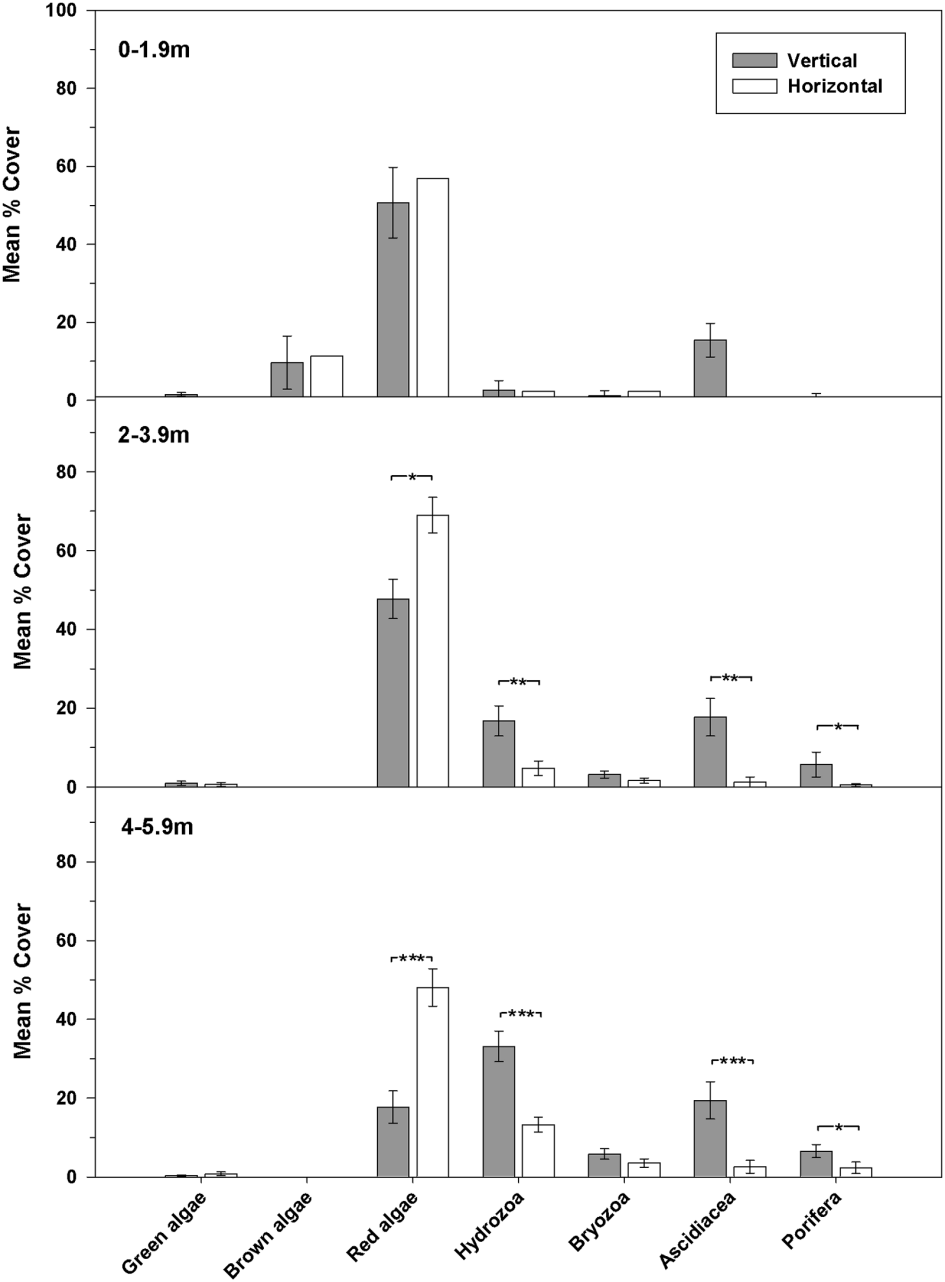
Epibenthic assemblages present on vertical and horizontal surfaces across the three depth ranges (0–1.9, 2–3.9, 4–5.9m) in 2012.

In 2012, differences in the coverage of red algae on vertical and horizontal surfaces were largely maintained at each depth range (Fig 6). Significant differences in coverage of the main faunal groups were also evident at middle and lower depths. Two-factor PERMANOVA performed on 2012 assemblages indicated significant differences between surface orientation and depths (Table 4). Pairwise comparison shows that in vertical assemblages, differences are evident between all depth groups (Table 5). Vertical surfaces from 2–5.9m are characterised by less macroalgae, increased presence of hydroids, bryozoans, ascidians, sponges and crustaceans. Horizontal assemblages at the two deepest depth ranges (2–3.9m and 4–5.9m) differ significantly. There is no significant interaction term between the two factors.

**Table 4:** Results of a two-factorial PERMANOVA on 2012 benthic data from the ASR across depths and types of surface orientation (vertical and horizontal).

Source	d.f.	S.S.	M.S.	Pseudo-F	p-value
**Depth**	2	9009.8	4504.9	6.6445	**0.0001**
**Orientation**	1	4393.2	4393.2	6.4798	**0.0004**
Depth*Orientation	2	1737.2	868.58	1.2811	0.2682
**Residuals**	55	37290	677.99		

**Table 5:** Results of pairwise comparison of benthic assemblages between surface orientation and depth ranges on the surf reef in 2012.

Depths	Orientation	t-statistic	p-value
**2–3.9m : 0–1.9m**	Vertical	2.0979	**0.0037**
**4–5.9m : 2–3.9m**	Vertical	2.5775	**0.0009**
**4–5.9m : 0–1.9m**	Vertical	3.7007	**0.0001**
**2–3.9m : 0–1.9m**	Horizontal	1.0483	**0.4007**
**4–5.9m : 2–3.9m**	Horizontal	1.9922	**0.0142**
**4–5.9m : 0–1.9m**	Horizontal	1.1032	**0.3491**

### Mobile species on the ASR

**Fig 7:**
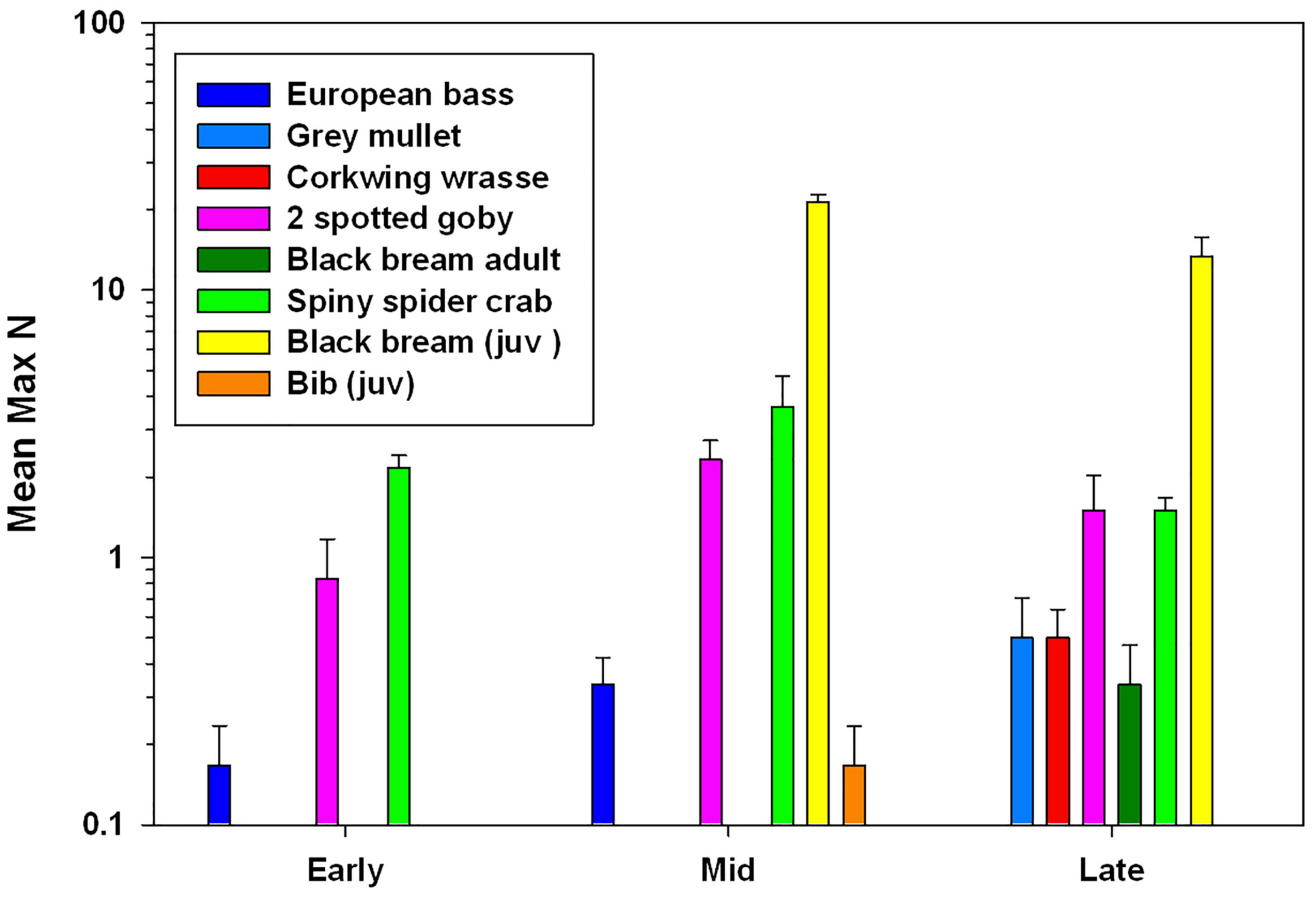
Mean abundance of species pairs of Pelagic adults (European bass, Grey mullet, Territorial adults (Corkwing wrasse, 2-spotted goby, Migratory (Black bream and Spiny spider crab) and Juvenile (Black bream and bib) in early, mid and late summer 2013. Data collected from BRUV surveys. Error bars show SE.

Adults of pelagic species bass (*D*.*labrax*) and mullet (*Liza* sp.) occurred in low numbers sporadically through the summer, which is consistent with anecdotal observations from divers and anglers. However Two-spotted gobies (*G*.*flavescens*) were present on the reef from early summer and could be seen swimming along the reef sides. Corkwing wrasse (*S*.*melops*), which are also known to exhibit territorial nesting behaviour, were only observed in late summer in these surveys. The spiny spider crab *M*.*squinado* was frequent on the ASR with MaxN of 17 observed in mid-summer, however although larger aggregations were observed around nearby piers, no ‘pods’ were observed on the ASR. Juvenile Black bream (*S*.*canthrus*) were very common in mid-late summer around the sides of the structure, where they gained shelter. ANOVA (Table 6) shows significant effects of behavioural/life-history “trait” and period through summer on species counts, and a significant interaction between the two factors. Counts showed no difference between species groups in early or late summer, but in mid-summer counts of juveniles were significantly higher than all other groups (territorial adults: p = 0.001; pelagic adults: p = 0.0001; migratory: p = 0.003) (Fig 7).

**Table 6:** Results of two-way ANOVA performed on species counts grouped by behavioural/life-history “trait” across each summer period from BRUV data.

Source	d.f.	S.S.	M.S.	Pseudo-F	p-value
Trait	3	707.7	235.907	8.476	**< 0.001**
Period	2	230.5	115.257	4.141	**< 0.05**
Trait*Period	6	485.8	80.970	2.909	**< 0.05**
Residuals	132	3673.8	27.832		

### Comparison with regional epibenthic communities

**Fig 8:**
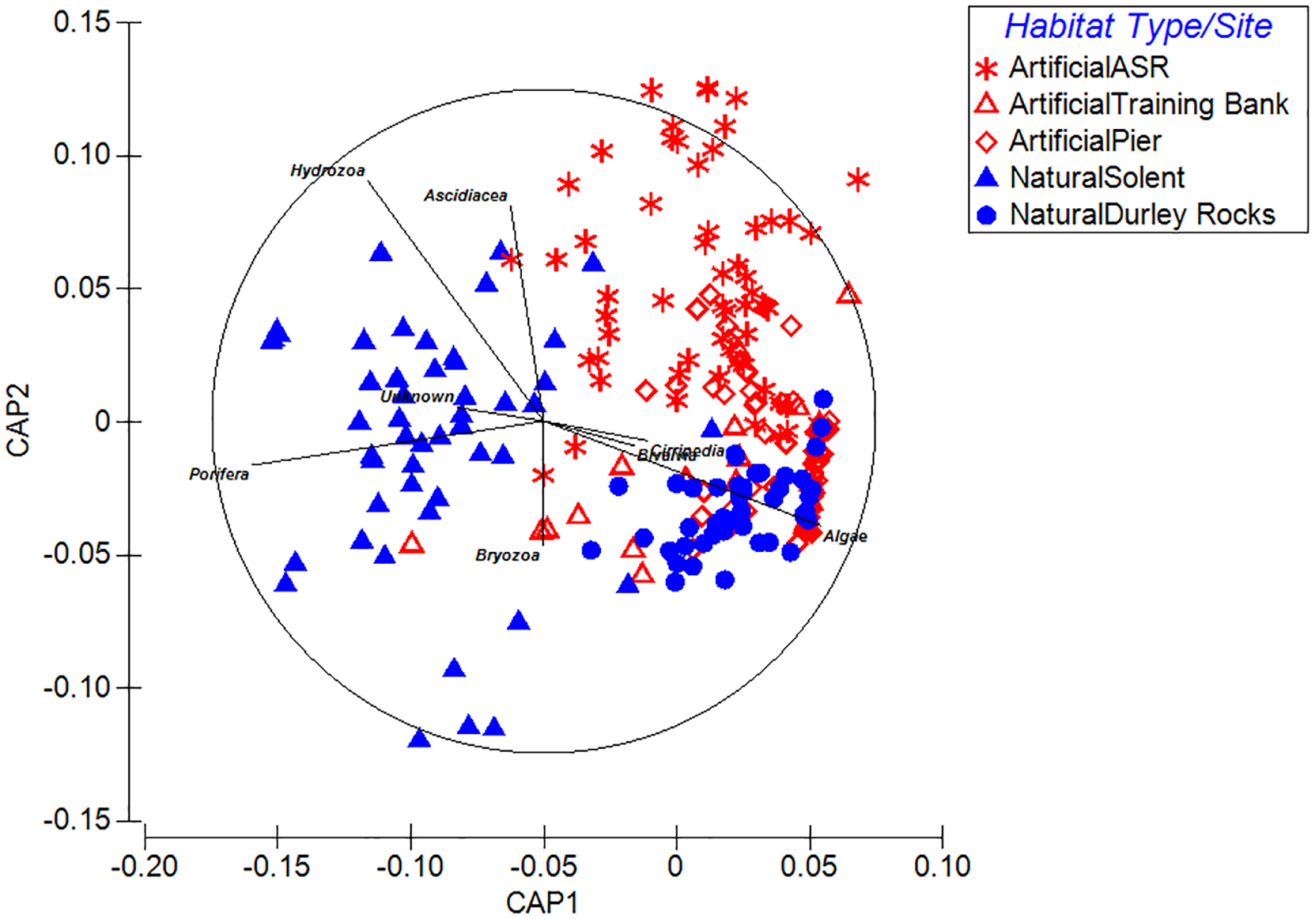
CAP ordination showing variability of samples across the different sites and habitats. Overlaid Spearman rank species correlations (> 0.4) species vectors. Discriminant analysis is based on 4 PCO axes accounting for 98% variability within the data.

ANOSIM shows significant differences in assemblages between sites (R = 0.468, p < 0.0001), with significant pairwise differences between all sites. CAP ordination procedure (Fig 8) indicates grouping of samples between each habitat type (trace statistic = 0.0001). Overlaid Spearman rank species correlations (> 0.4) indicate that assemblages at the natural reefs differed to the artificial habitats, including the ASR. The natural site in the Solent was characterised by higher abundances of sponges and hydroids whereas greater numbers of barnacles, bivalves and algae occurred at Durley Rocks in Poole Bay. The vertical surfaces of Boscombe Pier differed to the ASR as they were colonised by the kelp *Laminaria digitata*, barnacles (*Balanus crenatus*) and mussels (*Mytilus edulis*). The Poole Training Bank had a much greater coverage of sponges than the ASR, especially *Hymeniacidon perleve*.

## Discussion

### Colonisation of the Artificial Surf Reef

Variation in surface depth and orientation provided by different layers of sand bags creates a measure of complexity which has enabled an increasing coverage of different epibiota and functional groups over time. Within a metapopulation network, constituent species have potential to interact with neighbouring habitats and observed taxa indicate that colonisation is most likely the result of dispersal and recruitment of propagules from local hard substrata. Although natural reef habitat is sparse and discontinuous in the region, local structures within Poole Harbour and piers at Boscombe and Bournemouth have source populations that may have acted as stepping stones for the colonisation of algae, bryozoa and ascidians that have poor dispersal capabilities [80,81]. Timing of deployment of new substrata can have a significant impact on the rate and trajectory of succession due to variation in the availability of propagules in the water column [82,83] and it is most likely that the protracted duration of construction of the ASR would have resulted in variation in recruitment of taxa in different parts of the structure as they were completed and exposed.

Predictions that there would be increasing coverage of epibiota and replacement of early colonists over time were confirmed. Many colonising species overlap others and those lying directly below the image sampling point were not always identified, so some species coverage may have been underestimated or unrecorded. The shift from bryozoans to algal and ascidian dominated assemblages was pronounced and there was an increase in small gastropod consumers (*Rissoa parva*), amongst the algae and hydroids, which is consistent with Model 6 of Benedetti-Cecchi [22]. Bare patches observed on the ASR were mainly as a result of sand deposition and abrasion rather than grazing, as urchins and large invertebrate consumers are uncommon in Poole Bay. Initial colonisation of the ASR occurred during the construction phase and two years following completion (October 2011), over 55% of the substratum was covered by epibiota. This compares with 73% on horizontal slabs of cement stabilised pulverised fuel ash (PFA) from the nearby Poole Bay Artificial Reef (10m depth below Chart Datum) after 12 months (mean of upper and underside, May 1991–1992) [38]; 70% on subtidal concrete panels after 18 months in the Aegean Sea [19] and over 74% cover by mussels on new limestone blocks on low shore in the northern Adriatic over 12 months [84]. Algal communities can take several years to reach maturity, despite initially colonizing rapidly [85], and the growth and potential interaction of colonial ascidians and sponges is thought to be responsible for reductions in bryozoan coverage as the epibiotic community develops [86], as observed on the ASR.

The sequence of main groups of colonists was not dissimilar to nearby studies at the Poole Bay Artificial Reef [38,85 87] although its more rapid coverage compared to the ASR may have been due to a spring-time deployment or propagule supply. Distinct stages in the colonisation process have also been observed on other reefs [88–91], however across several locations and substratum types, the similarity of earlier stages have been found to be more random, due perhaps to varying levels of disturbance, compared to the more deterministic later stages [17,92]. It was not possible to obtain comparable annual detailed species inventories over the four year period as survey effort by volunteer divers varied between years. However the reef had become colonised by 71% of epibenthic and mobile species, recorded to date, by the first summer after completion in October 2010. Between 2011 and 2012, 25% of these species were still recorded as present however, notwithstanding species replacement during succession, this is likely to be an underestimate as the later inventories were not as thorough. Additional habitat complexity and resources afforded by the growth of algal assemblages and increased sedimentation of finer particles within narrow crevices between the sand bags are likely to have attracted later colonists.

At the base of the reef, growth of epibiota has been variable due to frequent abrasion by sand. Over time, natural physical and biological disturbances, varying spatially across habitats, are thought to be important in locally arresting the successional process in marine environments, with the resultant mosaic of patches exhibiting different successional stages [92]. At finer scales this was evident, yet was not quantified across the whole reef. However, from ROV footage and BRUV deployments, the area of deposit**e**d sand on the reef was observed to vary spatially and temporally across both horizontal and exposed vertical surfaces, where they could be partially buried. The deposition of sediments and potential sand-scour in mobile and dynamic habitats can have a profound effect on settlement, recruitment, growth and mortality of species associated with marine hard-substrata [93,94]. With the uniquely exposed location of the ASR surrounded by a mobile sandy sea bed, these disturbances are likely to continue to influence the colonisation of the structure and may determine the successional trajectory.

It has been shown that the influence of substratum type is often ephemeral and mostly affects the initial stages of colonisation [17,19], yet barnacles, which are normally frequent colonists on artificial structures and reefs [95–96] and influential in the establishment of intertidal communities [64], were notably absent on the ASR surface. Larval supply is unlikely to be limited as both *B*. *crenatus* and *Semibalanus balanoides* are abundant on local structures and tidal models reveal a strong easterly flow across the ASR from Poole Harbour [97]. The microstructural texture of non-woven geotextile materials can prevent barnacle settlement [98] yet in the longer term, the influence of the geotextile substratum on assemblages may be of lesser importance than other factors, such as disturbances and habitat complexity.

Structural complexity of artificial reefs is highly influential in determining type and diversity of benthic and mobile assemblages [33,55,99,100]. The ASR is a relatively simple structure and habitat complexity is created by variation in depth and surface orientation. Horizontal surfaces predominate on the ASR and were mainly colonised by red, green and brown algae, whereas vertical surfaces became dominated by ascidians and bryozoans. Vertical surfaces can create shade [101] yet may be exposed to more abrasion than other surfaces, depending on the local hydrodynamic environment. Although macroalgae is often prominent on shallow horizontal surfaces, especially in northern European seas, in other areas it is not always so [35] and there can be an interaction between light availability and the degree of sedimentation [39], which can be significant on parts of the ASR. The paucity of deep holes, fissures, and gullies on the ASR are limiting to larger fauna that require more shelter such as lobsters and crabs. These species are much more frequently observed at Durley Rocks and other nearby natural reefs where there are mixed sizes of crevices. Large kelps (e.g. *Laminariaceae* spp.) on Durley Rocks and Boscombe Pier can provide additional complex habitat and communities [102] and their current rarity on the ASR will limit species diversity. Measurement of spatial variation in structural complexity of the ASR, as has been determined at other sites [103] would have been very beneficial and assisted greatly with interpretation and analysis of benthic assemblages. Increasing depth has a significant impact on the type of assemblages present, with a reduction in macroalgae and an increasing prominence of invertebrates on both horizontal and vertical surfaces. A reduction in light intensity with depth combined with shaded vertical sides is possibly responsible [101], yet the effect of sedimentation would be worthy of further investigation, as would more detailed studies on the influence of inclined surfaces which were most similar to horizontal habitats at middle depths.

### Mobile species assemblages

Fish are particularly attracted to new structures including geotextile reefs [104]. The BRUV data combined with visual observations revealed an assemblage of mobile invertebrates and fish composed of species characteristic of both natural reefs, such as wrasse (*Symphodus melops*) and bib (*Trisopterus luscus*), and those associated with the sandy inshore habitat, including sand goby (*Pomatoschistus minutes*), hermit crab (*Diogenes pugulitor*) sand eel (*Ammodytes tobianus*) and dragonet (*Callionymus reticulatus*). These observations are consistent with others from isolated artificial reefs located on sandy habitats [55], which may present barriers for some species due to predation risk [105]. Although the influence of current strength and direction on the bait plume was minimised as far as possible, they can strongly influence data gathered [67,106], as can the type of bait [107,108], although oily fish, such as mackerel used in this study are preferred in sampling protocols [67,109]. Feeding behaviour and species interactions will influence which species do and don’t come to the bait [67,110] and cryptic or night foraging species may have been excluded. Large numbers of spider crabs around the bait did visibly attract other species as pieces escaped from the cage mesh and future studies combining this technique with un-baited cameras and diver visual census [55] would be beneficial to compare with these observations. A higher frequency of sampling throughout the spring and summer would have provided more detail on the use of the reef by mobile species with different behavioural and life history traits. This is particularly relevant to the sporadic records of pelagic species such as bass (*D*. *labrax*) and mullet (*Liza* sp.), that are able to traverse longer distances between isolated habitat, although this transient use and behaviour was confirmed anecdotally by SCUBA and local anglers. Species breeding territories were not confirmed; corkwing wrasse was seen with nesting material on the ASR in June 2010, but in 2013 the species was not recorded until after the main breeding season in September. Although Two-spotted gobies *G*. *flavescens* were prevalent and mostly likely resident, we could not confirm whether individuals seen and attracted to bait were territorial males [111]. The migratory spider crab *M*.*squinado* was very common during the summer, grazing on the vertical sides of the ASR, yet no large aggregations were observed which can be indicative of breeding, although they were seen elsewhere in the region. The abundance of juvenile (young of year) black bream (*S*. *cantharus*), which were seen in large numbers apparently gaining shelter around the edges of the ASR away from tidal streams, was of particular interest. This species which is of international conservation concern [77] and locally important for recreational anglers, is known to establish nests and territories in nearby sand and gravel habitats [78], making it vulnerable to exploitation. Spawn of several invertebrate species were seen attached to the ASR including annelida (Phyllodocidae sp.) squid (*Loligo* sp.), nudibranchia (*Facelina auriculata*) and whelk (*Buccinum undatum*). Clearly, future investigations should attempt to confirm use of the structure as a refuge for marine organisms at different life history stages.

### Comparison with other artificial and natural habitats

Subtidal rocky reef assemblages are uncommon in the region and the lack of control or reference sites can confound comparative studies with artificial reefs [48]. As observed on the ASR and known in other regions [42], depth variation can have a significant effect on assemblages of epibiota and fish. Therefore as some sampling locations were distant from the ASR and at different depths, an exact comparison of assemblages with the ASR cannot be made.

It was not possible to sample all sites in the same year so differences in assemblages could be due to inter-annual variation in recruitment, growth and disturbance.

In addition to differences in age [19,37,52] and habitat complexity [33,48,54], the location of artificial reefs and structures relative to natural reefs is a major source of variation in mobile assemblages, due in part to their isolation from contiguous natural reefs and species-specific differences in seasonal movements [56] and feeding traits [55]. The natural reefs in this study area are relatively small and isolated, so an increasing prevalence of new structures in the coastal environment may continually change the nature of habitat at a regional scale by facilitating movement and increasing patch connectivity.

The majority of the seabed within shallow inshore areas of Poole Bay consists of mobile sandy sediments of relatively low species diversity, so at a local scale (Boscombe), habitat formed of hard substrata has now increased. Yet all species so far recorded on the ASR are found in the locality, if not on all structures included in the study. Therefore, these novel structures may just alter the distribution pattern of locally abundant species rather than increasing species diversity at regional scales [112]. The youthful age of the ASR is undoubtedly an important factor affecting the comparison of benthic and mobile assemblages, and species have been found to accumulate with time [85,113,114]. With recruitment from the regional species pool and the influence of slow-growing and habitat-forming species such as sponges and kelps, convergence with natural reef communities may occur.

Of the 180 taxa recorded on the ASR, 11 are non-native, and more were recorded than at the other habitats, yet all have been previously observed in the region [87,115,116] (author’s observations). There is a concern that new structures with initially bare surfaces provide a spring board for the spread of NNS. The most prevalent NNS on the south coast of the UK is the slipper limpet *Crepidula fornicata*, which forms unattached chains on sedimentary seabeds and can also attach to hard surfaces. Its presence on the ASR is probably a result of storm wave action depositing adult chains on the reef rather than larval settlement, as the geotextile surface is likely to be unsuitable for direct attachment. Vertical surfaces on the surf reef support extensive coverage of ascidians, typically *Ascidiella aspersa* with a range of other species including the non-natives *Styela clava* and *Corella eumyota*. The latter was evident in 2010 but subsequently declined, reflecting author observations on an intertidal concrete outfall in the Solent. With opportunistic life history strategies, a combination of frequent disturbance and the proximity to ports and harbours could maintain high populations of NNS on the ASR. The structure may be a stepping-stone for dispersal of NNS, as well as native species associated with hard substrata. However with the prevalent occurrence of NNS in the region, it will be difficult to assign any negative impacts observed on natural reefs to a particular location or structure.

Justification for the construction of artificial reefs is often based on evidence that they will increase or restore local or regional species diversity and ecosystem services such as fisheries [12]. In northern temperate regions, artificial reefs are uncommon and their ecological and economic benefit has been rarely evaluated. The prevalence of juvenile *S*. *cantharus*, a commercially important species, is to date one of the more tangible benefits of the ASR and measurement of breeding success and temporal variation in abundance relative to other nearby habitats needs to be continued. Yet how, if at all, the ASR contributes to fish production is currently unclear. Measurements of fish using stereo-BRUV systems have been advocated to obtain quantitative data on growth of populations [55], which combined with more detailed investigations of feeding behaviour, to include Stable-Isotope Analysis, will provide valuable information on the use of these structures by different growth and life-history stages [117,118].

## Conclusions

Distinct stages in the colonisation of the ASR were observed which over four years has attracted a diverse and interesting assemblage of flora and fauna. There is variation in assemblages with depth and surface orientation that provides a measure of habitat complexity on an otherwise simple structure. Further experimentation to elucidate possible for reasons for this variation would be beneficial. Increasing both the depth and complexity of the ASR could be achieved by the deployment of rocks, bespoke artificial reef blocks or reef balls [119] at the seaward edges of the structure. This will also improve ecological functionality by providing a greater range of assemblage’s and refugia for different species. These modifications could also improve the quality of the SCUBA diving experience which may compensate for the poor surfing, thereby benefiting local tourism. It is important to continue to monitor the use of the current structure as assemblages develop and establish how it is being utilised by different species. This would be particularly valuable for breeding and migratory species and those of conservation concern and/or commercially important. An attempt to measure the wider impact of the structure on local sport angling and fisheries should also be investigated, as would monitoring the establishment of NNS on the ASR and nearby protected sites. In addition to significant assemblage dissimilarity with nearby habitats, the ASR has contributed to regional biodiversity by increasing the area of subtidal hard substrata and the size of local populations within the existing mixed pool of native and non-native species. Provided no negative impacts are detected on natural reefs, this may be considered a beneficial outcome of novel artificial structures.

## Appendix

**Table of Species recorded on Boscombe Artificial Reef (ASR). 2010–2014.**

Nomenclature according to Wold Register of Marine Species March 5^th^ 2017.

Species indicated by * are non-native

**KINGDOM ANIMALIA**

**Phylum Porifera**

Dysidea fragilis

*Halichondria* sp.

*Leucosolenia* sp.

Pachymatisma johnstonia

Polymastia mamilliaris

Suberites pagurorum

Sycon ciliatum

Sycon elegans

**Phylum Cnidaria**

Actinothoe sphyrodeta

Aglaophenia parvula

Aglaophenia pluma

Amphisbetia operculata

Anemonia viridis

*Bougainvillia*? *britannica*

Corymorpha nutans

Coryne eximia

Coryne muscoides

Dynamena pumila

Hydrallmania falcata

Laomedea flexuosa

Obelia dichotoma

Obelia longissima

Plumularia setacea

Rhizostoma pulmo

Sagartia elegans

Sertularia argentea

Sertularia distans

*Tubularia* sp.

Urticina felina

**Phylum Nemertea**

*Nemertea* sp.

**Phylum Platyhelminthes**

Prostheceraeus vittatus

**Phylum Annelida**

Ficopomatus enigmaticus*

*Harmothoe* sp.

Lanice conchilega

Nereididae

*Phyllodocidae* sp.

Sabella pavonina

*Spirobranchus* sp.

Spirobranchus triqueter

**Phylum Pycnogonida**

Achelia echinata

Ammothea hilgendorfi*

Endeis spinosa

*Nymphon* sp.

Pycnogonum litorale

**Sub Phylum Crustacea**

**Order Cirripedia**

Balanus crenatus

Hesperibalanus fallax

**Order Amphipoda**

*Gammarus* sp.

Caprella penantis

**Order Decapoda**

*Hippolytidae* sp.

Homarus gammarus

Cancer pagurus

Carcinus maenas

Diogenes pugilator

*Inachus* sp.

Liocarcinus marmoreus

Macropodia rostrata

Maja squinado

Necora puber

Pagurus bernhardus

Pagurus cuanensis

Pilumnus hirtellus

Pisa tetraodon

Pisidia longicornis

**Phylum Mollsuca**

**Class Gastropoda**

Buccinum undatum

Crepidula fornicata*

Doris pseudoargus

Doto millbayana

Eubranchus tricolor

Euspira catena

Facelina auriculata

Lacuna vincta

Nassarius incrassata

Ocenebra erinaceus

Rissoa parva

Tricolia pullus

Trivia monacha

**Phylum Mollusca**

**Class Bivalvia**

Mytilus edulis

Spisula solida

Venerupis corrugata

**Phylum Mollusca**

**Class Cephalopoda**

*Loligo* sp.

Sepia officinalis

**Phylum Bryozoa**

Amathia lendigera

Amathia citrina

Amathia imbricata

*Amathia* sp.

Bugulina flabellata

Bugulina turbinata

Chartella papyracea

*Crisia* sp.

Electra pilosa

Flustra foliacea

Membranipora membranacea

Scrupocellaria scruposa

Tricellaria inopinata*

**Phylum Chordata**

**Subphylum Tunicata**

Ascidiella aspersa

Ascidia mentula

Archidistoma aggregatum

*Botrylloides* sp.

Botryllus schlosseri

Clavelina lepadiformis

Ciona intestinalis

Corella eumyota*

Dendrodoa grossularia

*Molgula* sp.

Polycarpa scuba

Styela clava*

*Didedmnidae* sp.

Diplosoma listerianum

*Diplosoma*? *spongiforme*

Lissoclinum perforatum

**Phylum Chordata**

**Superclass Pices**

Ammodytes tobianus

Belone belone

Callionymus reticulatus

Symphodus bailloni

Symphodus melops

Ctenolabrus rupestris

Dicentrarchus labrax

Gobiusculus flavescens

Labrus bergylta

*Liza* sp.

Mullus surmuletus

Parablennius gattorugine

Platichthys flesus

Pollachius pollachius

Pomatoschistus minutus

Pomatoschistus pictus

*Pomatoschistus* sp.

Spondyliosoma cantharus

Syngnathus acus

Taurulus bubalis

Trisopterus luscus

Trisopterus minutus

**KINGDOM PLANTAE**

**Phylum Chlorophyta**

Cladophora pellucida

Codium fragile*

*Ulva* sp.

Ulva lactuca

Ulva linza

**Phylum Rhodophyta**

Asparagopsis armata*

Brongniartella byssoides

Calliblepharis ciliata

*Ceramium* sp.

*Chondria* sp.

Chondrus crispus

Chylocladia verticillata

Cryptopleura ramosa

Dilsea carnosa

Gastroclonium ovatum

Gracilaria bursa-pastoris

Gracilaria gracilis

*Gracilaria* sp.

Grateloupia turuturu*

Griffithsia corallinoides

Halurus flosculosus

*Heterosiphonia* sp.

Hypoglossum hypoglossoides

*Lomentaria* sp.

Membranoptera alata

Nitphyllum punctatum

Palmaria palmata

Phyllophora crispa

Plocamium cartilagineum

Polysiphonia elongata

*Rhodophyllis* sp.

Schottera nicaeensis

Sphaerococcus coronopifolius

Spiridia filamentosa

**KINGDOM CHROMISTA**

**Phylum Ochrophyta**

Cladostephus spongiosus

Colpomenia peregrina*

Dictyopteris polypodioides

Dictyota dichotoma

*Ectocarpus* agg.

Halopteris filicina

Halidrys siliquosa

Laminaria digitata

Saccorhiza polyschides

Sargassum muticum*

Scytosiphon lomentaria

*Sphacelaria* sp.

Stypocaulon scoparium

Taonia atomaria

## Supporting information

S1 File Available at: https://figshare.com/s/492b7689877e1e5c0e40. This Excel file contains data on the coverage of the main functional groups of the ASR (2009–2012), the coverage of main functional groups of other nearby habitats and the BRUV data for 2013.
(XLSX)
